# Mobile Phone Use Behaviors and Postures on Public Transportation Systems

**DOI:** 10.1371/journal.pone.0148419

**Published:** 2016-02-01

**Authors:** Huey-Wen Liang, Yaw-Huei Hwang

**Affiliations:** 1 Department of Physical Medicine and Rehabilitation, National Taiwan University Hospital and National Taiwan University College of Medicine, Taipei, Taiwan, ROC; 2 Institute of Occupational Medicine and Industrial Hygiene, College of Public Health, National Taiwan University, No 17, Xu-Zhou Road, Taipei, Taiwan, ROC; Tianjin University of Technology, CHINA

## Abstract

Mobile phones are common in our daily life, but the users’ preferences for postures or screen operating styles have not been studied. This was a cross-sectional and observational study. We randomly sampled passengers who used mobile phones on the Mass Rapid Transit (MRT) system in metropolitan Taipei. A checklist was used to observe their body postures and screen operating styles while sitting or standing. As a result, 1,230 subjects from 400 trips were observed. Overall, of all the passengers who were sitting, 41% of them were using mobile phones. The majority of the tasks involved browsing (84%) with their phones in a portrait orientation (93%). Different-hand holding/operating was the most commonly used operating style while sitting (46%) and same-hand holding/operating was the most common while standing (46%). The distribution of screen operating styles was significantly different for those sitting than for those standing and for different genders and age groups. The most frequently observed postures while sitting were having one’s trunk against a backrest, feet on the floor and with or without an arm supported (58%). As for the users who were standing, the both- and different-hands groups had a high proportion of arms unsupported, feet on the floor and either their trunk supported or not. In contrast, the same-hand group tended to have their trunk unsupported, were holding a pole or handstrap and had both feet on floor. Further studies are warranted to characterize the ergonomic exposure of these commonly used postures and operating styles, and our results will help guide the selection of experimental conditions for laboratory settings.

## Introduction

A mobile phone is no longer just a telephone and has become an integral part of modern living for many people. Mobile phone production rose from 450 million per year in 2011 to 984 million per year in 2013 [1], and more than 50% of the population in many western countries, as well as in Taiwan, own mobile phones [2, 3]. Approximately a decade ago, the major health concern with regard to mobile phone use was cancer, such as brain tumors or acoustic neuroma [4]. Currently, the exposure patterns have changed greatly, and mobile phones are not only used for talking and listening. The technical advancement of user interfaces through integrated touch-displays allows mobile devices to be controlled by the fingers, not styli or keyboards. Furthermore, the fast development of software and widespread of internet access widen the scope of applications far beyond communication. Therefore, the health concerns associated with mobile phone use have shifted to addictive use, psychological impact, safety issues and musculoskeletal symptoms [5–9].

The epidemiological data regarding the preferences of behaviors and postures among mobile phone users are important for evaluating their health effects. The changes in mobile phone usage are not only in how long people use them but also in why and how they use them. There are several ways to obtain this information on mobile phone use behaviors, and each bears different pros and cons. Questionnaires are used frequently and can include a large population, but their validity has been questioned [10]. Custom logging applications or activity recognition systems can obtain the frequency, duration, tasks and even locations of phone use and have provided valuable data in many areas, such as marketing, commerce, security, or social networking, etc. [11]. Nevertheless, they cannot register information regarding how people used the device to assess ergonomic postures or usability. Direct observation is the other option, allowing researchers to collect valid data regarding how people use mobile phones in certain situations. This has been used in some scenarios, including on university campuses, on the train or on the roadside [12–15].

There is a great diversity of typing styles and body postures among mobile phone users, which are of concern for several reasons. First, each posture is attributed to different ergonomic exposures and musculoskeletal risks [16, 17]. Finding the most frequently used postures can help to guide an experimental setup. Second, the design of applications or screen displays varies with screen operation preferences. One study in 2011 disclosed that 3.9% of the subjects used small electronic devices on train rides in Germany [12]. A recent study in the Netherlands found that 3.0% of cyclists used a phone, with the majority for operating (typing, texting) but not for calling, which differed from what had been observed 5 years earlier [14]. Another study on a university campus showed at least 6 typing styles for texting, with more than 85% of the subjects using either both hands to hold the phone and both thumbs to text, or their right hand to hold the phone and their right thumb to text [13]. In general, the research mentioned above has lacked details on use behaviors or has included a relatively small sample size.

We hypothesized that people used mobile phones on metropolitan rapid transportation (MRT) and that the screen operating styles and postures while using mobile phones would be different for sitting and standing. The goal of the current study was to evaluate the behaviors and preferences of postures and operating styles among passengers on the MRT system in the Taipei area through an observational study. The proportions, tasks, operating styles and body postures were analyzed.

## Materials and Methods

### Taipei Metro transportation system

The Taipei Metro system is the major mass rapid transit (MRT) system serving metropolitan Taipei. Since its opening in 1996, there have been 5 lines with 107 stations, operating on 129.2 km of revenue track. The trains operate from 6 AM to 1 AM and travel at a speed of 34 km/h. In 2014, the system carried an average of 1.86 million passengers per day and had outnumbered the number of bus passengers in mid-2013. The trains were either medium- (4 carriages) or high capacity (6 carriages). The chair measures are: seat height: 43 cm, seat depth: 36 cm and seat width: 46 cm. The backrest was reclined 15 degree with a vertical height of 42 cm from seat. None of the chairs had head rests or armrests.

### Sampling

This is a cross-sectional and observational study. The observed population included MRT passengers who were using mobile phones during the observation. We excluded those who used wheelchairs, brought walking devices, or had visible neuro-musculoskeletal disorders that might affect their postures. The study lasted 5 weeks starting on April 13th, 2015, and the observation was only conducted between 7AM and 9 PM on each day. Rush-hours or not was by the definitions from the Taipei Metro Company.

First, we randomly sampled 10 time periods (by hour) each week and 2 adjacent stations for each time period. Observation was conducted for 4 round-trips between these 2 stations, so a total of 80 trips were selected per week. For each trip, the observation was restricted to a designated area that was randomly pre-selected. The carriage was divided into 5 or 6 areas, depending on design of the space. Then, we randomly selected the carriage number and the area of the carriage for each trip. Only the mobile phone users in this area were observed. We also counted the number of passengers who sat in the designated area but did not use mobile phones.

This study was approved by the Research Ethics Board of National Taiwan University Hospital, and written informed consents were waived under the criteria that no direct contact or intervention be made with the participants and no personal information collected.

### Observation checklist for mobile phone use

We observed both body postures and screen operating styles of mobile phone users. For body posture, an observation checklist was adapted from a previous study [12] for standing and sitting (Table 1). For sitting, no observation of the head was performed because the seats provided no head support. For arm positions, resting on body parts (laps) or personal belongings (such as hand bags or backpacks) were not distinguished, and both were registered as supported. As for operating styles, we recorded which hand(s) people used to hold the device or operate the screen. A pilot observation on MRT was used to verify the above definitions. During the observation period, the observers boarded the chosen carriage at the preceding station to occupy a proper position for observation. When the passengers were settled in their seats or positions when the train departed the selected station, the observation began. It took approximately 30 to 40 seconds to complete the checklist for one subject. Afterwards, we categorized the screen operating styles into 4 groups: using both hands to hold and operate (both-hand group), using the same hand to hold and operate (same-hand group), using different hands to hold and operate (different-hand group) and others (unclassified). The unclassified group included talking on the phone, putting the phone on one’s lap and holding it without doing any screen operations. In addition, device-related parameters were observed, including the device direction (portrait or landscape) and tasks (browsing, texting, playing games, talking on the phone, or listening).

**Table 1:** Features of sampled trips and subjects.

Variables	Trips (N = 400)	Subjects (case = 1,230)
Time*		
Rush hours	143 (35.8)	517 (42.0)
Semi-rush hours	19 (4.8)	58 (4.7)
Non-rush hours	238 (59.5)	655 (53.3)
Weekdays		
Monday	63 (15.8)	186 (15.1)
Tuesday	64 (16.0)	207 (16.8)
Wednesday	72 (18.0)	228 (18.5)
Thursday	57 (14.3)	171 (13.9)
Friday	96 (24.0)	264 (21.5)
Saturday	24 (6.0)	90 (7.3)
Sunday	24 (6.0)	84 (6.8)
Routes		
Wenhu line	63 (15.8)	198 (16.1)
Tamsui-Xinyi line	95 (23.8)	290 (23.6)
Songshan-Xindian line	92 (23.0)	269 (21.9)
Zhonghe-Xinlu line	71 (17.8)	230 (18.7)
Bannan line	79 (19.8)	243 (19.8)
Time in a day		
before 10 a.m.	130 (32.5)	384 (31.2)
11 a.m.- 3 p.m.	141 (35.3)	411 (33.4)
after 4 p.m.	129 (32.3)	435 (35.4)

*The definition of rush hour/semi-rush hours/non-rush hour was according to MRT company.

Last, we also registered the gender and estimated age range of the users. A pilot test for inter-rater reliability of the checklist between the 2 observers was conducted with 49 subjects, and the *Kappa* agreement of all parameters was above 0.9, except for age (0.78).

### Statistical analysis

The descriptive data are expressed as either a mean value or a frequency, and the statistical analysis was performed by SAS statistical software (SAS 9.3 for Windows, 2012). χ^2^ or Fisher’s exact test was used to compare categorical data. Significant differences were defined at 0.05.

## Results

A total of 400 trips were sampled, with 1,230 observations completed (Table 2). Overall, of all the passengers who were sitting, 41% of them were using mobile phones and d 1.6%, using tablet computers. During 11 trips, no passengers in the selected area used a mobile phone. Approximately 36% of trips and 42% of subjects were sampled during non-rush hours. The distribution of trips and subjects in the morning, daytime and evening were of similar proportions. Eighty-eight percent of the trips were sampled on weekdays, with Fridays having the highest percentage of sampled users. The majority of mobile phone users were young to middle-aged, and more than 80% were less than 40 years old (Table 3). Approximately two thirds of the users were sitting, and females accounted for more than 60% of all subjects. Approximately 84% of the subjects were browsing on their mobile phones upon observation and 93% used the devices in a portrait orientation. Approximately 78% of the subjects used one hand to hold their mobile phones, and using the left hand only was more common than using the right hand. Several of the above features were different between subjects who were sitting and those who were standing while using their phones. Those subjects sitting tended to be older, female and more likely to use both hands to hold their phones than those standing.

**Table 2:** Coding for body postures.

Description of sitting postures		Coding	Description of standing postures		Coding
Trunk	Free from backrest	1	Trunk	Free from support	1
	Against backrest	2		Back against wall/pole	2
	Lounging (slumped back)	3	Arms	Free from support	1
Arms	Free from armrests	1		Holding a pole	2
	Wrist/forearm supported	2		Holding a handstrap	3
	Elbow supported	3	Legs	Free, both feet on floor	1
Legs	Free, both feet on floor	1		Single-foot stance	2
	Knee crossed	2			
	Other	3			

**Table 3:** Comparison of demographic and device-related features between subjects sitting or standing to use smartphones.

Variables	All (n = 1,230)	Sitting (n = 817)	Standing (n = 413)	p value
Age				
<20 years	91 (7.4)	52 (6.4)	39 (9.4)	<0.0001
20–40 years	919 (74.7)	579 (70.9)	340 (82.3)	
40–60 years	205 (16.7)	174 (21.3)	31 (7.5)	
>60 years	15 (1.2)	12 (1.5)	3 (0.7)	
Gender				
Male	454 (36.9)	274 (33.5)	180 (43.6)	0.001
Female	776 (63.1)	543 (66.5)	233 (56.4)	
Tasks				
Browsing	1,038 (84.4)	686 (84.0)	352 (85.2)	0.72
Texting	99 (8.0)	66 (8.1)	33 (8.0)	
Gaming	46 (3.7)	33 (4.0)	13 (3.1)	
Talking	32 (2.6)	20 (2.4)	12 (2.9)	
Listening	15 (1.2)	12 (1.5)	3 (0.7)	
Device orientation				0.45
Portrait	1,147 (93.3)	765 (93.6)	382 (92.5)	
Landscape	83 (6.7)	52 (6.4)	31 (7.5)	
Holding device				
Both hands	270 (22.0)	195 (23.9)	75 (18.2)	<0.0001
Right hand	283 (23.0)	159 (19.5)	124 (30.0)	
Left hand	673 (54.7)	459 (56.2)	214 (51.8)	
Nil	4 (0.3)	4 (0.5)	—	
Screen operation				
Both hands	121 (9.8)	75 (9.2)	46 (11.1)	<0.0001
Right hand	753 (61.2)	536 (65.6)	217 (52.5)	
Left hand	219 (17.8)	110 (13.5)	109 (26.4)	
Nil	137 (11.1)	96 (11.8)	41 (9.9)	

A total of 25 and 9 combinations of body postures were observed for sitting and standing, respectively. For both sitting and standing, only 5 combinations of body postures had a frequency higher than 5% (Table 4), and these 5 top postures accounted for 78.3% and 96.7% of all observed sitting and standing postures, respectively. For passengers sitting and using their mobile phones, the most frequent body posture was having one’s trunk against the backrest, wrist/forearm supported and both feet on the floor (posture 221), followed by a similar posture but free from armrests (posture 211). For passengers standing and using their phones, the most frequently observed body posture was having one’s trunk against a wall/pole, arms free from support and both feet on the floor (posture 211), followed by a similar posture, but with the trunk free from support (posture 111).

**Table 4:** Top 5 body postures observed in standing or sitting.

Sitting (N = 817)		Standing (N = 413)	
Body postures	N (%)	Postures	N (%)
221	258 (31.6)	211	127 (30.8)
211	217 (26.6)	111	95 (23.0)
231	73 (8.9)	212	68 (16.5)
222	51 (6.2)	131	66 (16.0)
213	41 (5.0)	121	43 (10.4)

As for operating styles, the different-hand group accounted for the highest proportion (40%), followed by same-hand group (30%) (Table 5). The majority of the different-hand group used their left hand to hold the phone and operated the screen with their right hand, irrespective whether they were sitting or standing. More people in the single-hand group used their right hand while sitting. The distribution was also significantly different in terms of whether users were sitting or standing, their gender and age group (Table 6), but not in terms of time in a day. The standing users had a higher proportion of using the same hand to hold/operate, while the sitting users more often used a different hand for holding versus operating. Additionally, female subjects and the older group tended to use different hands to hold and operate their mobile phones more than males and the younger group.

**Table 5:** Hands used for holding and screen operation.

Operating styles	All (n = 1,230)	Sitting (n = 817)	Standing (n = 413)
Both-hand	270 (22.0)	195 (23.9)	75 (18.2)
Different-hand	486 (39.5)	372 (45.5)	114 (27.6)
Holding with right hand	35 (2.8)	24 (2.9)	11 (2.7)
Holding with left hand	451 (36.7)	348 (42.6)	103 (24.9)
Same-hand	369 (30.0)	180 (22.0)	189 (45.8)
Right hand	204 (16.6)	109 (13.3)	95 (23.0)
Left hand	165 (13.4)	71 (8.7)	94 (22.8)
Unclassified	105 (8.5)	70 (8.6)	35 (8.5)

**Table 6:** Comparison of screen operating styles for using mobile phones among subjects with different demographic features and sitting/standing.

Variables	Both-hand N = 270	Same-hand N = 369	Different-hand N = 486	UnclassifiedN = 105	p value*
Posture					<0.0001
Standing	75 (18.2)	189 (45.8)	114 (27.6)	35 (8.5)	
Sitting	195 (23.9)	180 (22.0)	372 (45.5)	70 (8.6)	
Gender					<0.0001
Male	111 (41.1)	180 (48.8)	129 (26.5)	34 (32.4)	
Female	159 (58.9)	189 (51.2)	357 (73.5)	71 (67.6)	
Age					<0.0001
<20 years	28 (10.4)	38 (10.3)	14 (2.9)	11 (10.5)	
20–40 years	217 (80.4)	299 (81.0)	329 (67.7)	74 (70.5)	
40–60 years	23 (8.5)	31 (8.4)	134 (27.6)	17 (16.2)	
>60 years	2 (0.7)	1 (0.3)	9 (1.9)	3 (2.9)	
Time in a day					0.08
before 10 a.m.	85 (31.5)	130 (35.2)	135 (27.8)	34 (32.4)	
11 a.m.- 3 p.m.	101 (37.4)	114 (30.9)	169 (34.8)	27 (25.7)	
after 4 p.m.	84 (31.1)	125 (33.9)	182 (37.4)	44 (41.9)	

*Comparison by χ^2^ test or Fisher’s test as appropriate.

**Fig 1:**
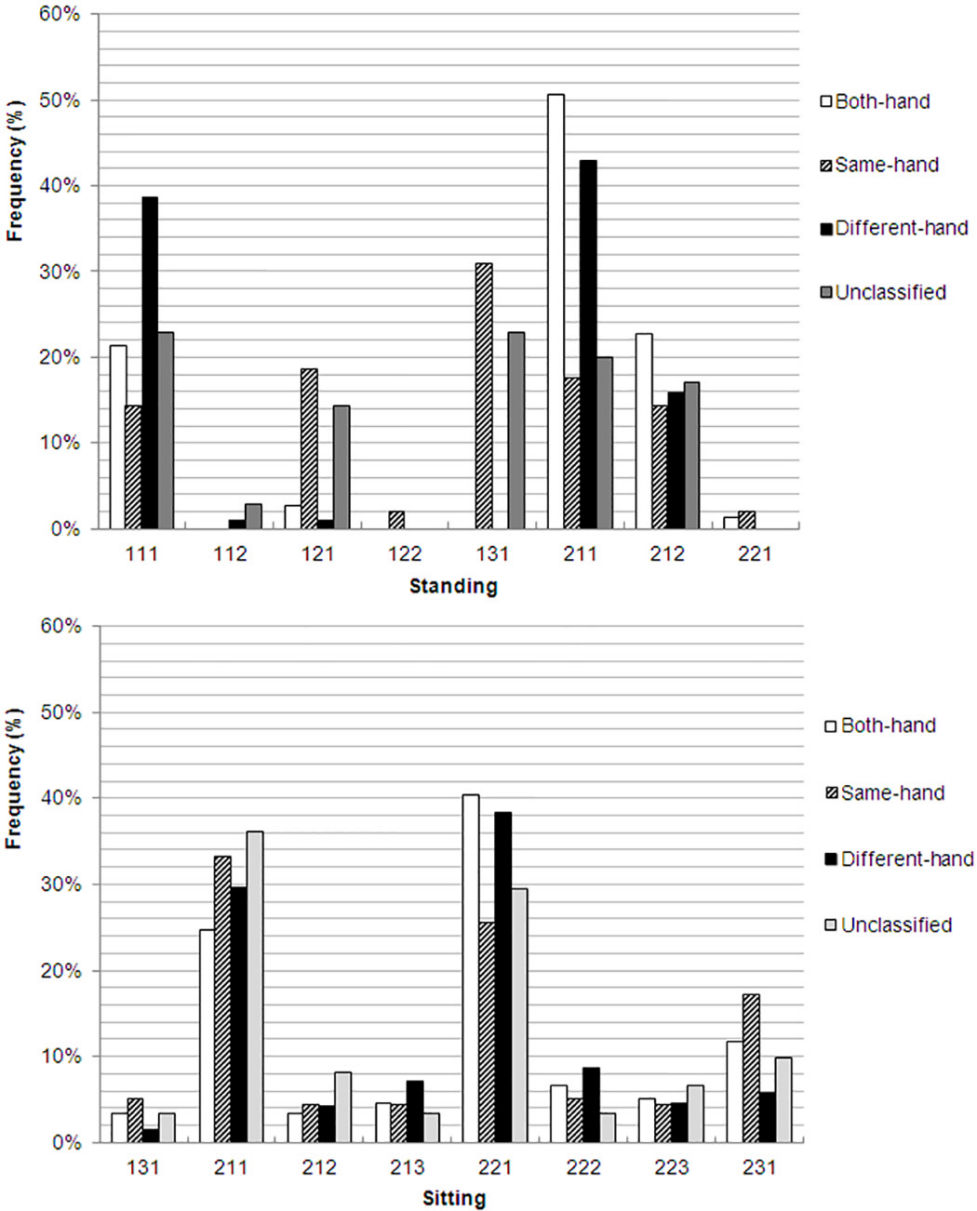
The postures used by phone users with 4 screen operating groups durings standing and sitting.

Fig 1 shows the distribution of the combinations of postures in the four operation style groups while sitting or standing. Postures 211 and 221(trunk against backrest, feet on floor and with or without arm support) were most commonly observed across the 4 styles while sitting. For users standing, the both- and different-hands groups used a high proportion of postures 111 and 211 (trunk supported or not, arm free and both feet on the floor). In contrast, the same-hand group tended to use postures 131 and 121 (trunk unsupported, holding a pole or handstrap and both feet on floor) most frequently.

## Discussion

### Research questions

This observation study characterized several important features of mobile users on the MRT. First, a high percentage (41%) of passengers who were sitting used mobile phones. Browsing was the most frequent activity, and holding the device in the portrait orientation was very common (93%), whether the user was sitting or standing. Operating styles and body postures, however, were different between passengers who were sitting and standing. These results confirmed the high dependence on mobile phones and that the great variability of postures and styles when people use their mobile phones on public transportation. The study’s implications and limitations warrant further discussion.

So far, this was the first observational study focusing on the postures and operating styles of mobile phone users on public transportation. Most of the previous studies observed users on-the-go with a focus on safety issues. The few studies related to mobile phone use behaviors during waiting or stationary situations have been conducted on train journeys, leisure situations or on campuses [12, 13]. They were either convenience or small samples. Our choice of the MRT passengers had some advantages in terms of study design. First, it provided opportunities to access a sample closer to the general population. A survey in Taiwan in 2013 showed that people use smartphones most frequently at home, followed by at work, restaurants and on public transportation (79%) [18]. Of these places, public transportation is the only option where direct observation and a large sample size are possible. It also had the advantages of standardized settings, seats and lighting, and these environmental factors all influence postures and behaviors. It could also help to obtain a real-world scenario in a field study because of differences in mobile phone use behaviors in the laboratory and on the MRT [19]. Finally, because the MRT systems are common in many rural areas, our research design could be used as a standard protocol to compare the mobile phone use patterns across different countries or cities.

### Popularity of mobile phone usage

The percentage of passengers sitting on the MRT using their mobile phones was 41%. This was much higher than that obtained in an earlier study that observed people’s postures during sitting activities on train journeys or semi-public/leisure situations in 2013 [12]. Only 3.9% and 6.3% of the subjects used a small electronic device in the above two conditions. One possible reason is the fast growth of mobile phone users and dependence on phones everywhere in the past few years [20]. In addition, Cultural differences and the sample’s demographics are also possible explanations, although there are no other studies to support these possibilities. Unlike using mobile phones while driving or walking, there was less of a safety issue related to using phones while sitting or standing in public areas, such as the MRT. However, they did reflect the high intensity of phone use. Problematic or addictive use of mobile phones has been associated with certain demographic features and personal traits [21, 22]. For example, women are more likely to become addicted to mobile phones than men [21]. Nevertheless, the demographics of the current sample, largely young- to middle-aged and with more female subjects, may be representative of only the population who uses the MRT.

The mobile phone activities engaged in most on the MRT were browsing, whether sitting or standing, while talking on the phone comprised a surprisingly low percentage (2.6%). This finding supports the claim that mobile phones are used much more as a multi-activity portal than as a phone. A previous report in Taiwan showed that subjects used their smartphones for entertainment (94%), communication (86%) and staying informed (75%) [18]. Another survey of students and university staff also showed the time spent using a mobile phone was on average 2.23 hours browsing the internet and 2.13 hours listening to music, watching videos and taking pictures [16]. They also found significant associations between the time spent browsing the internet or using a mobile device and pain in the neck and upper limbs. The above findings raised the problem regarding the extent of mobile phone use and the potential health effects. Moreover, some researchers have highlighted the impact of the environmental differences in usability tests of mobile phones in a laboratory or on the MRT [19]. The noise level of the environment, the motion in the MRT setting, the lack of privacy, the increased effort needed to perform tasks and the additional stress/nervousness were some of the subjects’ concerns. Further studies are required to evaluate whether using phones on the MRT bears additional hazards.

### Screen operation styles

People use several different styles to operate the screens of their mobile phones. Overall, the different-hand group made up the highest proportion (40%), closely followed by the same-hand group (30%) and then by the both-hands group (22%). These proportions are different from a previous study, which revealed the most frequently used styles to be both hands holding/texting or the right hand holding/texting in college-age subjects on a campus [13]. In our study, the majority of passengers who were sitting used their right hand to hold the phone and their left hand to operate screen. It was observed that 46% of standing passengers used the same hand to hold/operate the phones (same-hand group), with approximately an equal proportion using their right or left hand. The difference in the results may be attributed to tasks (browsing *vs*. texting). Moreover, we assumed that people choose their operating styles based on comfort and performance. From the prospective of biomechanical loads, same-hand typing has a higher muscle load for the wrist extensors of the finger/thumb flexors than both-hands typing because of the increased effort needed for stability while holding the phone and operating it simultaneously [23–25]. It also rendered a slower texting speed [25]. However, for people standing, it remained the preferred posture, possibly due to the need to use the other hand to carry things or hold poles/handstraps and the low demand on typing while browsing on the mobile phone. As for the difference between the different-hands and both-hands groups, there was no head-to-head comparison of the muscle activities or joint angles for these 2 styles. Our results showed a high preference for different-hands operating, especially in the older-age group and females. As for using the same hand for holding/operating, the both-hand group should have higher muscle loads and a better texting performance. When the people are engaged in activities requiring less texting, such as browsing, they are apt to taking on a comfortable posture. We also suspected the size of a mobile phone influences the preference. One laboratory test on 20 subjects found that more subjects used their right index finger in combination with other digits to text as the phone screen size increased [24].

### Body postures and operating styles while sitting

In our results, combinations of body postures were more frequent for those sitting than for those standing (25 *vs*. 9), but only 5 combinations had a frequency higher than 5% for each (Table 4). For sitting phone users, the body posture of the highest frequency was having one’s trunk against a backrest, wrist/forearm supported and both feet on the floor (posture 221), followed by a similar posture but free from armrests (posture 211). In contrast, posture 211 (without considering the head posture) was the leading observed posture for people using small and larger electronic devices (high-level activities) during the train journeys or semi-public/leisure situations in an earlier study [12]. From the viewpoint of comfort, it seems to be more reasonable for people to choose an appropriate support of their body parts to reduce their musculoskeletal loads. Our results revealed that more than 60% of the phone users had their upper limbs supported while sitting, especially in the both-hands group. The rationale was supported by the reduced muscle activities of the trapezius and biceps brachii for phone users with their elbows supported on their laps [25]. What was different from the designed posture in laboratory settings was that quite a few people had their elbows or wrists supported on their personal belongings on the MRT, instead of in their laps.

### Body postures and operating styles while standing

The postures of mobile phone users who were standing have been observed in the laboratory and the field [13, 26]. Nevertheless, the postures used while standing on the MRT are likely to be different from standing on a steady floor, as was documented by a study related to postural control at sea and on land [27]. Control of standing is more complicated than sitting and involves the action of the muscles of the whole body. The external environment and the individual’s actions influence the equilibrium that preserves one’s posture. There was no study available to characterize a standing posture while using mobile phones on the MRT, but our results illustrated some accommodating strategies. Most of the phone users chose to use some kind of support while standing, including leaning on walls or holding straps/poles because of the benefits of support to postural adjustment [28]. Only 23% of them used their phones without any support. The distribution of the screen operating styles was significantly different among the different body postures in terms of the trunk and arms. When the users were not supported (posture 111 and 112), the different-hands group had the highest proportion, likely because of the convenience of shifting to one-hand holding if necessary.

### Study limitations

There are several issues in considering the generalization of the results. We used random sampling to ensure a representative population of MRT passengers, but it did not represent the general population. The demographics showed a mostly young and middle-aged population, and the older population or those who used other methods of transportation were underrepresented. Second, we were not able to obtain more detailed information on the activities people were engaging in (browsing the internet, watching videos, etc.) on their mobile phones or their demographic features (eyesight, exact age, anthropometry), which might have a significant impact on operating styles or postures. Finally, this cross-sectional design could not capture a dynamic change or the effect of longer-duration phone use. For example, people may briefly check their phone or change their behaviors while the train is speeding up or approaching a station.

## Conclusion

This was the first study observing on the postures and operating styles of mobile phone users on public transportation. A high proportion of MRT passengers used mobile phones, and the most frequently activity they engaged in was browsing. Body postures and typing styles were closely related. Different-hand holding/operating was the most commonly used operating style during sitting, while same-hand holding/operating was the most commonly used during standing. We also observed the most frequently chosen body postures for phone users on the MRT. Further studies are warranted to characterize the ergonomic exposure of these commonly used postures and operating styles, and our results can help to guide the selection of experimental conditions in laboratory settings.
